# Render EEG-Based Brain–Computer Interfaces Calibration-Free: Trade Space for Time in EEG Decoding

**DOI:** 10.1109/OJEMB.2026.3667029

**Published:** 2026-02-23

**Authors:** Maohua Liu, Shi Wang, Wenzhe Cui, Shihui Zhang, Liqiang Zhao, Fred R. Beyette

**Affiliations:** School of Electrical and Computer EngineeringUniversity of Georgia 1355 Athens GA 30602 USA; School of Mathematics and Information Science and TecnhologyHebei Normal University of Science and Technology 165079 Qinhuangdao 066004 China; School of Information Science and EngineeringYanshan University 26472 Qinhuangdao 066004 China; UII America Inc. Burlington MA 01803 USA; Stony Brook University 12301 Stony Brook NY 11790 USA; School of Information Science and EngineeringYanshan University 26472 Qinhuangdao 066004 China; School of Mathematics and Information Science and TecnhologyHebei Normal University of Science and Technology 165079 Qinhuangdao 066004 China

**Keywords:** BCI, EEG decoding, calibration, deep learning

## Abstract

*Goal:* Electroencephalogram-based brain-computer interfaces (EEG BCIs) have broad applications in neurorehabilitation, clinical assessment, and assistive technologies. However, their practical deployment is severely limited by subject-specific calibration, which requires time-consuming data collection and model retraining for each user, significantly reducing usability. This reliance on calibration arises from the conventional “one-model-fits-all” strategy: “relying on a single general model to handle all data complexity like subject variability. When its limited generalization falls short, time must be spent on calibration to adapt the model.” *Methods:* To address this limitation, we propose a trade-space-for-time strategy for calibration-free EEG decoding: “Instead of adapting one model to every user, we maintain a pool of compact models, including a general model and multiple biased models, where each biased model specializes in decoding a specific type of subject pattern. For a new input, the system automatically selects the most suitable model based on data characteristics, enabling instant adaptation without retraining.” Compact deep learning models make this design feasible by allowing fast switching and low storage cost, which would be impractical with large-scale architectures. *Results:* Experiments on multiple public EEG datasets show that the proposed strategy achieves performance comparable to within-subject decoding: slightly higher in one dataset (0.7672 vs. 0.7601), nearly identical in another (0.7568 vs. 0.7572), and marginally lower in a third (0.8804 vs. 0.8888). *Conclusions:* These results demonstrate that our approach effectively eliminates calibration while preserving accuracy, providing a practical and scalable alternative for EEG BCIs. The framework also has potential applications in other neuroimaging modalities such as fMRI and fNIRS.

## I. Introduction

Eeg BCIs are non-invasive systems that enable direct interaction between the brain and external devices, with applications in neurorehabilitation, neurological assessment, and assistive technology [1], [2], [3]. However, EEG signals vary greatly across and within users due to anatomical, physiological, and mental factors, making frequent calibration indispensable for maintaining performance of EEG BCIs [4], [5], [6], [7], [8]. This calibration is time-consuming, fatiguing, and a major barrier to practical adoption [9], [10], [11], [12], [13].

To overcome calibration, recent work has explored improving model generalization, data augmentation, and meta-learning, while some aim for fully calibration-free EEG BCIs.

Improving model generalization is key to reducing or eliminating calibration in EEG BCIs, mainly by learning representations that generalize well across subjects [14], [15]. Methods include subject-invariant feature learning to suppress subject-specific noise [16], [17], latent space transformations to align EEG features between subjects [18], and domain adversarial training to remove subject-specific identity and promote consistency [19], [20]. Other strategies involve selecting data common to all subjects [21], using encoder-sharing networks [22], applying Riemannian manifolds to unify heterogeneous features [23], [24], and leveraging Transformer architectures for long-range relationship modeling [25], [26], [27].

Data augmentation simplifies calibration by reducing the need for extensive labeled data. Conventional methods like cropping and scaling enhance performance across datasets [28], while channel reflection increases spatial variability to boost robustness [29], [30]. Generative adversarial networks (GANs) have shown strong potential, such as ERP-WGAN generating realistic ERP signals to enrich limited datasets [31], and cGAN or DCGAN synthesizing diverse EEG patterns to improve cross-subject generalization [32]. Synthetic EEG data have also been shown to support accurate decoding, demonstrating promise for practical and user-friendly BCIs [33].

Meta-learning enables rapid adaptation to new subjects with minimal labeled data by learning strategies or initializations that support fast personalization. Model-Agnostic Meta-Learning (MAML) adapts effectively with only a few gradient steps [34], while meta-transfer learning refines adaptation through flexible embeddings or transformations [35], [36]. These methods are especially useful in few-shot EEG settings, reducing fine-tuning requirements, though some calibration remains necessary [37].

Calibration-free strategies have also been explored. Data alignment aligns EEG features across subjects to remove individual differences [38], [39], enabling a single model to handle multiple users without calibration [40], [41], though accuracy often declines [42]. Unsupervised models adapt online without labeled data by leveraging EEG structure [43], and reinforcement learning supports continuous adaptation through exploration and exploitation, allowing immediate use without pre-calibration [44].

Despite their individual strengths, these strategies have yet to deliver truly calibration-free EEG BCI systems. Generalization improvement, data augmentation, and meta-learning have made significant progress in reducing calibration demands, but none can fully eliminate it. Calibration-free approaches do make it but fall short in accuracy, limiting their practical adoption. A key reason for this limitation is that most existing methods still rely on the traditional one-model-fits-all (OMFA) paradigm, in which a single general model is expected to accommodate wide inter-subject and inter-session variability. When such generalization inevitably fails, calibration becomes the default remedy. This tight coupling between limited generalization and mandatory retraining reveals a structural weakness in the OMFA design, rather than a deficiency of any single algorithm. As long as EEG decoding is constrained by this paradigm, calibration remains unavoidable. This paper proposes a more practical solution to break from this OMFA tradition. Consider this alternative: after each calibration of the general model, we back up its calibrated weights. By maintaining a cache pool of recently used (i.e. calibrated) versions of the general model, the system can efficiently retrieve the most appropriate one when similar data are encountered, eliminating the need for repeated calibration. This is essentially a trade-space-for-time (TSFT) strategy. It is effective for different EEG modalities including MI [45], SSVEP [46], [47] and P300 [48]. It can also be used to any other neuroimaging modality like MRI [49], [50], fMRI [51], [52] and RGB image [53], [54]. The main contributions of this paper are as follows:

1. Proposes a new direction for designing calibration-free EEG BCIs, with the potential to completely eliminate calibration.
2. Introduces the TSFT strategy, achieving cross-subject decoding accuracy comparable to within-subject decoding.
3. Explores a novel application of compact deep learning models in EEG decoding.
4. Presents a simple and effective method for designing a signature for each compact model.

The rest of the paper is organized as follows: Section II introduces related works. Section III details the proposed TSFT and experimental setup, Section III reports and discusses the validation results, Section IV concludes the study and Section V talks about future work.

## II. Related Works

TSFT is mainly based on the continuous simplification of the decoding model. The emergence of the ultra-compact model is the fundamental premise of TSFT and also the most important related work. TSFT was not feasible prior to the development of compact models for EEG decoding. Traditionally, general models were large and parameter-heavy, making it impractical to maintain a large number of models within a single decoding task. The high computational and memory demands posed significant challenges. However, EEG decoding models are now becoming increasingly compact. Early architectures like CNN1 (2010) had more than a million parameters [55], and complexity peaked with CNN-R (2015), which has about 20 million parameters [56]. Later models like DeepConvNet (2017) [57] improved accuracy with simpler structures with no more than 0.2 million parameters. But it was EEGNet (2018) [58] that marked a turning point, using depthwise separable convolutions to reduce parameters to the hundreds while maintaining the best performance. This is the first time the parameter count dropped below one thousand. More recent models such as SepConv1D (2021) [59], BCNN (2024) [60], and P300MCNN (2024) [61] further minimized the size, with P300MCNN achieving state-of-the-art results using just 161 parameters and 315 FLOPS, as shown in Table I. P300MCNN is generally the simplest model, having the fewest parameters and lowest computational cost (FLOPs) consistently across all datasets. This indicates that P300MCNN is extremely lightweight and computationally efficient compared to deeper and more complex networks, making it well suited for real-time EEG decoding and deployment on resource-constrained systems without sacrificing practicality. Also, P300MCNN is generally the best one in training epochs, traing time (TT) and inference time (IT), as shown in Table II. We detail the complexity of P300MCNN as it forms the core of the proposed TSFT framework, and the complexity analysis of the other components is provided separately in another section.

The move to compact models makes TSFT practical. The motivation of TSFT is to take advantage of this ultra-compactness by organizing compact models into a collaborative swarm. This swarm allows rapid adaptation to diverse users or states through real-time selection of the most suitably configured model, eliminating retraining.

**TABLE I:** Number of Parameters (Param) and Floating-Point Operations Per Second (FLOPS) of Different Architectures Across Datasets [59], [61]

Measure	CNN1/UCNN1	CNN3/UCNN3	CNN-R	DeepConvNet	ShallowConvNet	BN3	EEGNet	OCLNN	FCNN	SepConv1D	P300MCNN
(a) P300-LINI											
Param	1,036,922	1,031,009	19,848,098	140,627	12,162	44,633	1,474	1,842	2,477	225	161
FLOPS	2,073,642	2,061,816	39,683,214	278,976	24,088	89,304	2,801	3,653	4,950	443	315
(b) BCI Comp. II											
Param	787,502	781,067	16,445,794	175,677	104,402	39,649	2,338	14,706	19,973	1,361	723
FLOPS	1,574,802	1,561,932	32,878,606	349,076	208,568	79,394	4,529	29,381	39,942	2,715	2,858
(c) BCI Comp. III											
Param	1,207,502	1,201,067	21,950,818	177,677	105,362	47,841	2,434	14,898	2,885	1,405	891
FLOPS	2,414,802	2,401,932	43,888,654	29,765	210,488	95,778	4,721	353,076	5,766	2,803	3,140
(d) BNCI Horizon											
Param	1,036,942	1,031,011	19,848,290	141,877	15,362	44,673	1,506	2,290	3,301	265	197
FLOPS	2,073,682	2,061,820	39,683,598	281,476	30,488	89,386	2,865	4,549	6,598	523	387

**TABLE II:** Epochs (E), Train Time (TT), and Inference Time (IT) Obtained by the Architectures on Each Dataset [59], [61]

Architecture		P300-LINI	BCI Comp. II	BCI Comp. III	BNCI Horizon
CNN1	E	97 $\pm$ 33	68 $\pm$ 8	65 $\pm$ 6	71 $\pm$ 14
	TT	5.33 $\pm$ 1.74	8.08 $\pm$ 1.39	59.66 $\pm$ 9.6	5.79 $\pm$ 1.29
	IT	0.04 $\pm$ 0.004	0.06 $\pm$ 0.013	0.24 $\pm$ 0.015	0.05 $\pm$ 0.006
UCNN1	E	88 $\pm$ 27	74 $\pm$ 26	66 $\pm$ 12	76 $\pm$ 24
	TT	4.89 $\pm$ 1.46	8.8 $\pm$ 3.01	63.93 $\pm$ 15.76	6.27 $\pm$ 1.98
	IT	0.04 $\pm$ 0.004	0.06 $\pm$ 0.013	0.24 $\pm$ 0.014	0.05 $\pm$ 0.006
CNN3	E	111 $\pm$ 37	86 $\pm$ 28	85 $\pm$ 29	93 $\pm$ 31
	TT	5.25 $\pm$ 1.75	9.68 $\pm$ 3.28	76.1 $\pm$ 28.19	6.56 $\pm$ 2.13
	IT	0.04 $\pm$ 0.003	0.06 $\pm$ 0.01	0.24 $\pm$ 0.013	0.05 $\pm$ 0.004
UCNN3	E	114 $\pm$ 42	78 $\pm$ 13	72 $\pm$ 16	87 $\pm$ 30
	TT	5.41 $\pm$ 1.97	9.01 $\pm$ 1.84	68.42 $\pm$ 20.46	5.87 $\pm$ 1.93
	IT	0.04 $\pm$ 0.003	0.06 $\pm$ 0.009	0.24 $\pm$ 0.011	0.05 $\pm$ 0.004
CNN-R	E	61 $\pm$ 2	167 $\pm$ 29	89 $\pm$ 29	64 $\pm$ 2
	TT	12.46 $\pm$ 0.49	47.25 $\pm$ 6.57	148.38 $\pm$ 45.75	18.68 $\pm$ 0.67
	IT	0.07 $\pm$ 0.01	0.09 $\pm$ 0.026	0.32 $\pm$ 0.026	0.09 $\pm$ 0.014
DeepConvNet	E	122 $\pm$ 40	79 $\pm$ 10	122 $\pm$ 25	106 $\pm$ 24
	TT	13.33 $\pm$ 4.15	26.41 $\pm$ 2.88	276.22 $\pm$ 58.13	18.16 $\pm$ 3.97
	IT	0.11 $\pm$ 0.007	0.13 $\pm$ 0.013	0.37 $\pm$ 0.013	0.12 $\pm$ 0.010
ShallowConvNet	E	177 $\pm$ 29	144 $\pm$ 45	95 $\pm$ 40	157 $\pm$ 33
	TT	16.57 $\pm$ 2.69	63.41 $\pm$ 18.85	281.23 $\pm$ 116.77	25.12 $\pm$ 5.29
	IT	0.06 $\pm$ 0.011	0.09 $\pm$ 0.015	0.37 $\pm$ 0.018	0.06 $\pm$ 0.010
BN$^{3}$	E	113 $\pm$ 21	77 $\pm$ 4	71 $\pm$ 3	95 $\pm$ 9
	TT	4.04 $\pm$ 0.7	8.8 $\pm$ 1.31	71.08 $\pm$ 10.78	5.06 $\pm$ 0.56
	IT	0.07 $\pm$ 0.001	0.09 $\pm$ 0.007	0.27 $\pm$ 0.009	0.08 $\pm$ 0.002
EEGNet	E	200 $\pm$ 3	166 $\pm$ 30	175 $\pm$ 30	198 $\pm$ 7
	TT	17.18 $\pm$ 0.5	53.69 $\pm$ 7.28	360.66 $\pm$ 62.81	27.67 $\pm$ 1.13
	IT	0.08 $\pm$ 0.005	0.10 $\pm$ 0.005	0.31 $\pm$ 0.008	0.09 $\pm$ 0.005
OCLNN	E	199 $\pm$ 5	129 $\pm$ 41	87 $\pm$ 11	161 $\pm$ 26
	TT	4.55 $\pm$ 0.28	11.88 $\pm$ 2.84	75.69 $\pm$ 17.95	5.87 $\pm$ 0.99
	IT	0.04 $\pm$ 0.002	0.05 $\pm$ 0.003	0.22 $\pm$ 0.005	0.04 $\pm$ 0.002
FCNN	E	197 $\pm$ 7	89 $\pm$ 21	98 $\pm$ 11	132 $\pm$ 12
	TT	3.74 $\pm$ 0.21	5.71 $\pm$ 1.26	53.88 $\pm$ 7.23	4.04 $\pm$ 0.38
	IT	0.02 $\pm$ 0.001	0.04 $\pm$ 0.002	0.17 $\pm$ 0.014	0.03 $\pm$ 0.001
SepConv1D	E	199 $\pm$ 5	104 $\pm$ 14	90 $\pm$ 12	183 $\pm$ 24
	TT	5.34 $\pm$ 0.32	10.61 $\pm$ 2.01	80.02 $\pm$ 14.7	8.22 $\pm$ 1.14
	IT	0.03 $\pm$ 0.002	0.05 $\pm$ 0.003	0.22 $\pm$ 0.009	0.04 $\pm$ 0.002
P300MCNN	E	60 $\pm$ 2	10 $\pm$ 1	10 $\pm$ 1	40 $\pm$ 2
	TT	5.33 $\pm$ 0.32	11.01 $\pm$ 2.08	89.62 $\pm$ 16.42	7.71 $\pm$ 1.07
	IT	0.03 $\pm$ 0.002	0.05 $\pm$ 0.003	0.21 $\pm$ 0.009	0.04 $\pm$ 0.002

## III. Materials and Methods

**Fig. 1.**
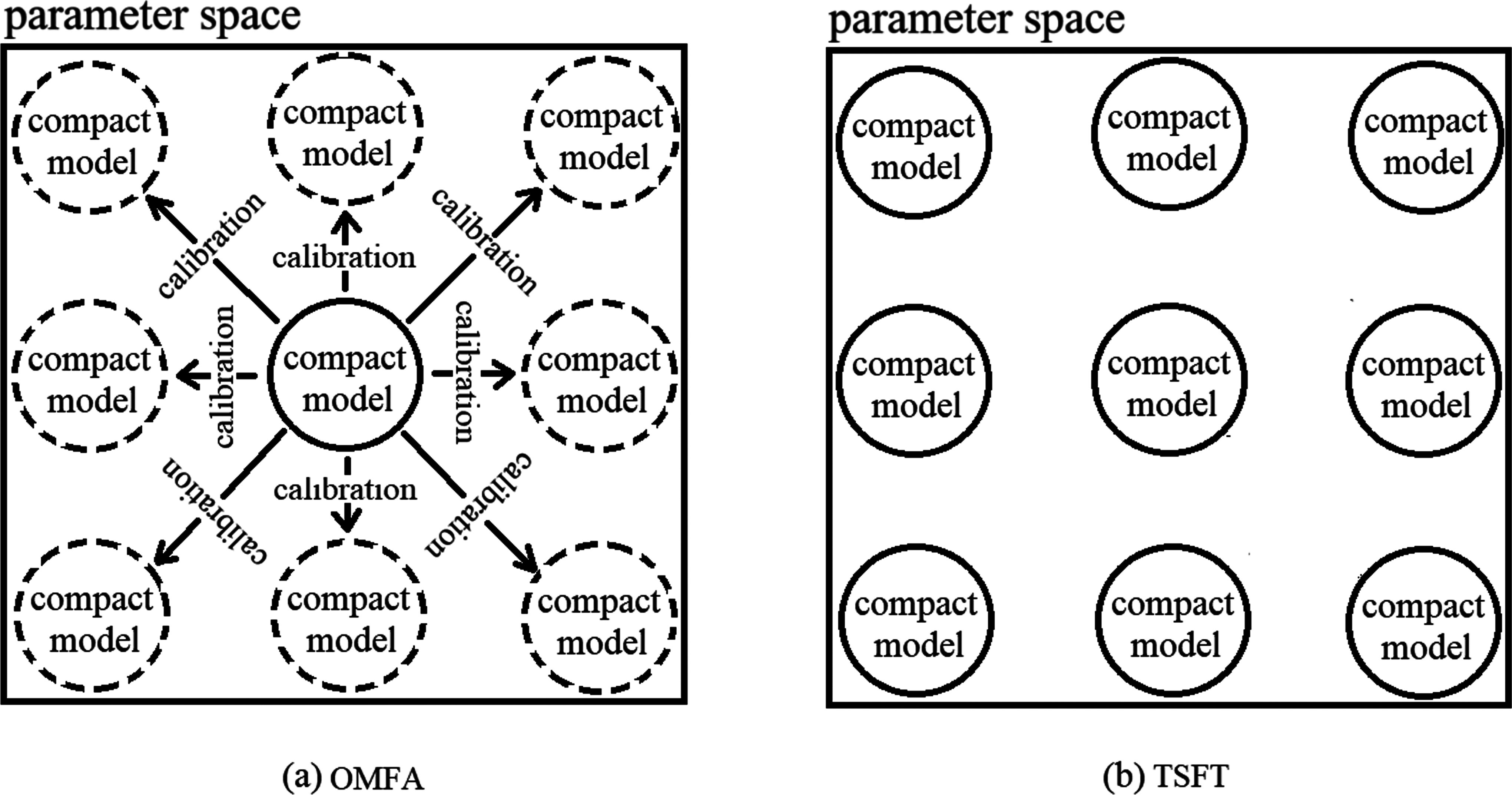
OMFA vs. TSFT.

The shift toward compact decoding models brings both challenges and opportunities. Their main limitation is a reduced capacity of a single compact model to manage high subject variability without calibration. However, with only a few hundred parameters, compact models can be scaled into swarms that are efficient to store and easy to update. Fig. 1 compares OMFA and TSFT. In OMFA (Fig. 1(a)), the square shape represents the parameter (or model) space which is large while the only solid circle in the center represents the only compact model which is also a general model. A solid circle indicates a model that physically exists in the system. Each dashed circle represents a fine-tuned or biased version derived from the single compact model. A dashed circle does not permanently exist; it is temporarily created through fine-tuning and discarded after decoding is completed. The arrows show calibration, meaning that calibration is required to get the biased model from the only compact model. All biased models start from the only compact model and then shift in different directions through calibration to fit different users. This shows that the system depends heavily on per-subject calibration, and each subject ends up with a separate dashed model located at a different place in the parameter space. As a result, much time will be spent on fine-tune or calibration. In TSFT (Fig. 1(b)), the square shape represents the parameter (or model) space and there is no dashed circle anymore which means no time is required to spend on calibration to get new biased models to fit different users. Instead, each solid circle represents a compact model that is already well positioned in parameter space and does not require calibration. The models are learned in advance and distributed evenly, so each one works well for certain users without finetune. This illustrates a calibration-free system: instead of calibrating model to fit new user, the system directly selects the most suitable pre-trained model for a user. In summary, the left diagram shows a calibration-dependent approach, where models must be calibrated for each user. The right diagram shows a calibration-free approach, where suitable models already exist. No calibration is needed—switching directly to the appropriate biased model is sufficient. TSFT avoids expensive calibration and makes the system easier to use and scale. This section details the proposed TSFT, along with the datasets, experimental setup, and evaluation methodology.

### A. TSFT

**Fig. 2.**
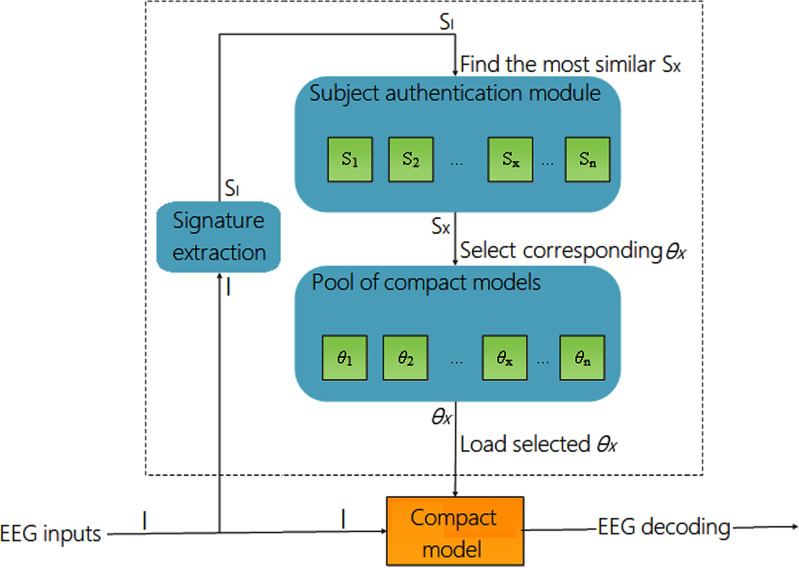
Implementation of TSFT.

Fig. 2 illustrates the implementation of TSFT. Given an input EEG segment $I$, the system first extracts a compact representation, or signature, denoted as $S_{I}$, which characterizes the subject’s EEG pattern. The *Subject authentication module* then compares $S_{I}$ to a library of pre-stored signatures $(S_{1}, S_{2},\ldots, S_{n})$ and identifies the most similar one, $S_{x}$. Based on this match, the corresponding model parameter set $\theta _{x}$ is retrieved from the *Pool of compact models* and loaded into the decoding network. The key innovation lies in the module enclosed by the dashed box, which enables real-time reconfiguration of the decoding model. Unlike traditional calibration, which requires labeled data and fine-tuning, the proposed framework performs subject adaptation by directly replacing the model parameters without any additional training. This mechanism allows instantaneous switching between subject-specific models, enabling efficient personalization even for unseen users. This approach is made practical by advances in compact deep learning models, which require minimal storage and support fast parameter loading. Such rapid reconfiguration would be infeasible with large, monolithic architectures. The following sections detail TSFT’s main components: the subject authentication module, signature design, compact model pool, and compact model architecture.

**Fig. 3.**
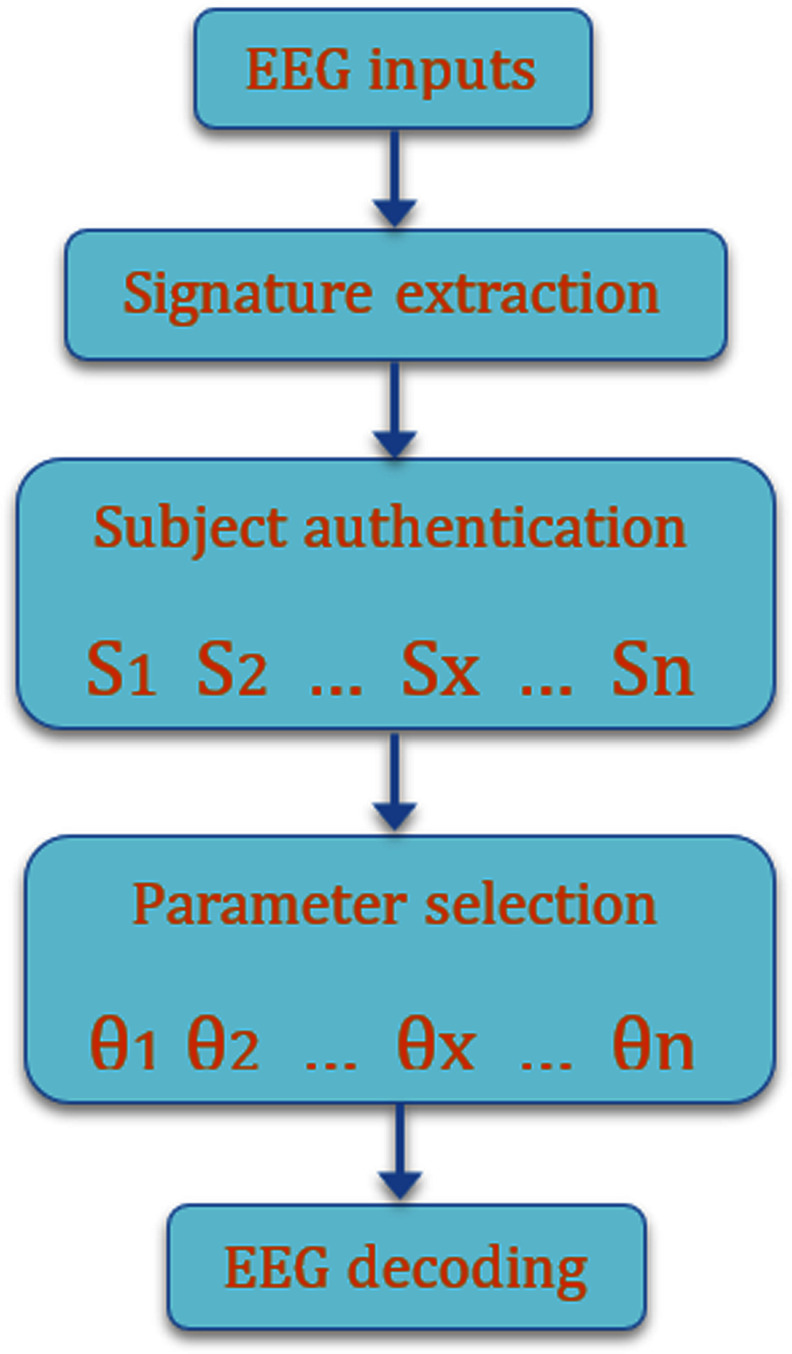
Flowchart of TSFT.

As a supplementary explanation of TSFT, a simple flowchart is provided in Fig. 3. First, raw EEG signals are provided as input to the system. Next, a signature extraction module computes a subject-specific representation from the EEG data. This signature is then passed to a subject authentication module, which compares it with stored subject signatures and identifies the most similar subject $S_{x}$. Based on the identified subject, the system performs parameter selection by loading the corresponding model parameters $\theta _{x}$. Finally, the selected parameters are used by the compact model to perform EEG decoding and generate the output.

Subject authentication module. This module selects the most appropriate compact model for decoding by comparing the signature of the input data with those of pre-stored models. An effective signature should be simple, distinctive, and predictive, with its similarity indicating how well the corresponding model matches the current data. Signatures may be vectors, matrices, statistical features, or outputs from existing subject authentication algorithms [62], derived from either the models or their training data. EEG-based authentication methods, capable of distinguishing users from few or even single epochs, are well suited for this purpose [62].

For TSFT verification, each model’s signature was defined as the standard deviation of each EEG channel in its training data. Given subject $S_{1}$ with $N$ epochs, $T$ time points, and $C$ channels, we first average the $N$ epochs, then compute the per-channel standard deviation across time in this average epoch. These values form a signature vector $(\sigma _{1}, \sigma _{2},\ldots, \sigma _{C})$. Signature similarity is measured as the distance $D$ between vectors using (1). If multiple signatures yield the same distance, the system defaults to the compact general model in the pool to handle ambiguous cases.

\begin{equation*} D=\sum _{i=1}^{C}(\sigma _{i} - \sigma _{i} ^{\prime })^{2} \tag{1} \end{equation*}

Pool of compact models. This module stores a set of compact models and dynamically loads the one most suited for EEG decoding. The required number of models in a real BCI system depends on performance targets, storage capacity, and user diversity, and stored models can be updated as needed. The lightweight nature of compact models makes maintaining a large collection feasible. In our experiments on public datasets, we trained one model per subject plus an additional compact general model. This pool was sufficient to achieve cross-subject decoding performance comparable to within-subject accuracy.

Compact model. We used P300MCNN for the P300 dataset and EEGNet for other EEG types [58], [61]. P300MCNN is the smallest architecture achieving state-of-the-art P300 decoding, while EEGNet, though slightly larger, generalizes well across diverse EEG data. Both contain only a few hundred parameters and can be further reduced through pruning or other optimizations. Full architectural details are provided in [58], [61].

### B. Datasets

We evaluated TSFT using three publicly available EEG datasets: the Motor Imagery (MI) dataset [63], [64], the Error-Related Negativity (ERN) dataset [65], [66], [67], [68], and the P300-LINI dataset [69], [70]. These are widely used in the BCI community for their structured designs, diverse subject pools, and clearly defined EEG features, making them ideal benchmarks for cross-subject decoding.

### C. Experimental Setup

To evaluate TSFT, we designed experiments comparing its performance with the within-subject decoding paradigm, tailoring the implementation to the characteristics of each dataset.

**Fig. 4.**
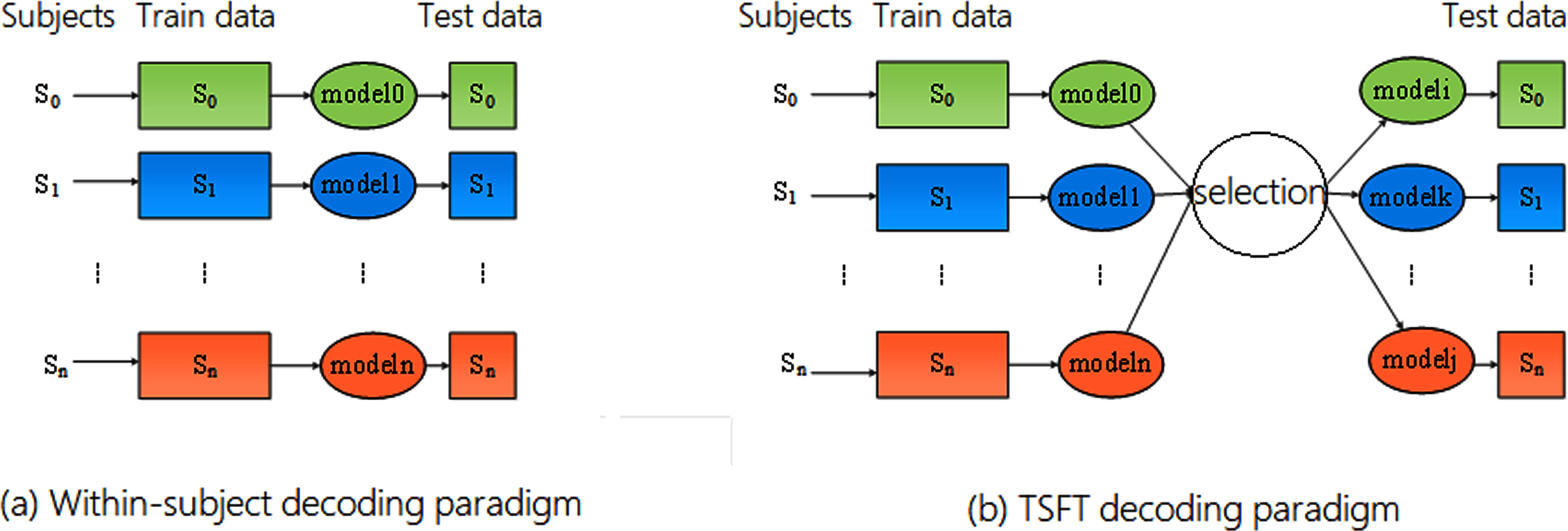
Within-subject decoding paradigm vs. TSFT decoding paradigm.

For the MI dataset, which specifies the first three sessions for training and the last two for evaluation, we adhered strictly to this setup. As shown in Fig. 4, we compared the within-subject decoding paradigm with TSFT. The ERN dataset has a similar restriction: each of the 16 subjects has five sessions, with 60 epochs in each of the first four sessions and 100 in the last. We used all 240 epochs from the first four sessions for training and the 100 from the last for testing.

The P300 dataset lacks predefined splits, so we applied $RepeatedStratifiedKFold$ to enable systematic evaluation. Data were divided into five folds, each serving once as the test set while the remaining four were used for training. This process was repeated three times with different random splits, producing 15 runs in total, while preserving the original class distribution.

Fig. 4 compares two different paradigms of using models for EEG decoding. In within-subject decoding (Fig. 4(a)), each subject has their own model. A model is trained using the data from one subject and is only tested on that same subject. This means every subject requires a separate training process. This approach usually gives good performance because it doesn’t need to worry about subject variability. In TSFT decoding (Fig. 4(b)), a model is still trained for each subject, but during testing the system no longer requires knowing the subject’s identity. Instead, a selection module compares the new EEG input with stored subject signatures and automatically chooses the most suitable model from the model pool. The selected model may not be the one originally trained on that subject, but the one whose learned patterns best match the current input. This allows the system to adapt across subjects without retraining and enables fast, calibration-free decoding. In short, the left approach relies on one model per person, while the right approach relies on selecting the best model for the input data, making TSFT more flexible, scalable, and practical.

All experiments were conducted on a Windows Enterprise N system with an Intel Core™i7-8850H CPU (2.60 GHz) and 16 GB RAM. Models were implemented in Keras with TensorFlow 2.10.1.

## IV. Results and Discussion

This section is organized into three subsections, each presenting the results and discussion for one dataset (MI, ERN, and P300). For each dataset, we report signature distance and model matching results, model matching time, and F1 score comparisons.

### A. MI Dataset

The core step in TSFT is selecting the most suitable model to decode the current test data by identifying which model’s signature most closely matches that of the test data. In our experiments, each model’s signature is a vector of channel-wise standard deviations $\sigma$ computed from its subject’s training data. Model matching thus becomes a comparison of $\sigma$-based distribution distances between test and training data, with the smallest distance indicating the best match.

Table III shows how similar each subject’s test data is to every subject’s training data using a distance metric. Each row is a test subject, and each column is a training subject. A smaller number means the two data distributions are more similar, and therefore the corresponding model is more suitable for decoding. For most subjects, the smallest distance appears on the diagonal (e.g., 0$\rightarrow$ 0, 1$\rightarrow$ 1, 2$\rightarrow$ 2), meaning each subject’s data is most similar to their own training data. This confirms that a subject’s own model is usually the best match. However, two special cases appear. For subject 3, the distances to subjects 0, 1, and 3 are all equally small (0.08). Because no single subject is clearly the best match, a general model (G) is selected instead. For subject 4, the closest match is not their own data, but subject 5’s training data (0.34 vs. 0.54), so the system selects model 5 rather than model 4. Overall, the table demonstrates that the proposed matching strategy usually selects the correct subject-specific model, while also handling ambiguous or atypical cases by choosing a general or more suitable alternative model.

**TABLE III:** Distribution Distances Between Test Data and Train Data and the Matching Results. (Note: “g” is “general Model”.)

Test dataTrain data	0	1	2	3	4	5	6	7	8
0 $\qquad \rightarrow$ 0	0.02	0.04	0.50	0.09	0.78	1.66	0.30	0.16	0.43
1 $\qquad \rightarrow$ 1	0.03	0.02	0.51	0.02	0.67	1.80	0.16	0.17	0.36
2 $\qquad \rightarrow$ 2	0.34	0.25	0.04	0.37	0.32	1.32	0.84	0.75	1.20
3 $\rightarrow$ G	0.08	0.08	0.35	0.08	0.85	1.95	0.24	0.16	0.38
4 $\qquad \rightarrow$ 5	2.17	1.92	1.41	2.51	0.54	0.34	3.61	3.44	4.39
5 $\qquad \rightarrow$ 5	3.35	3.05	3.10	4.00	1.79	0.22	5.33	4.89	6.06
6 $\qquad \rightarrow$ 6	0.19	0.28	0.76	0.08	1.41	3.17	0.01	0.02	0.05
7 $\qquad \rightarrow$ 7	0.13	0.15	0.66	0.10	1.22	2.59	0.17	0.07	0.19
8 $\qquad \rightarrow$ 8	0.28	0.38	1.04	0.17	1.68	3.44	0.03	0.03	0.02

Table IV shows how long the system takes to decide which model should be used for decoding EEG data. Each column represents a matching case where the system compares EEG data from one subject (on the left number) to another subject’s model (on the right number). For example, “0 $\rightarrow$ 0” means subject 0 is matched to their own model, while “3 $\rightarrow$ G” means subject 3 is matched to a general model. The numbers in the table are the time required (in microseconds) to compute the distribution distance and select the most suitable model. Most matching processes take 104.9 microseconds, which is extremely fast. Even the slowest case (“0 $\rightarrow$ 0”) takes only about 209.6 microseconds, and the average is 120.2 microseconds, showing that the model selection step adds almost no delay. Overall, the table demonstrates that TSFT can find a suitable decoding model very quickly, making real-time, calibration-free EEG decoding practical.

**TABLE IV:** Time Spent for Calculating Distribution Distances and Finding Matches (Unit:microseconds, “g” is “general Model”)

0 $\rightarrow$ 0	1 $\rightarrow$ 1	2 $\rightarrow$ 2	3 $\rightarrow$ G	4 $\rightarrow$ 5	5 $\rightarrow$ 5	6 $\rightarrow$ 6	7 $\rightarrow$ 7	8 $\rightarrow$ 8	Average
209.6	119.8	109.5	110.3	110.0	105.6	106.1	104.9	105.8	120.2

Table V compares the decoding performance (F1 score) of two methods: the within-subject decoding paradigm and the TSFT decoding paradigm. For most subjects (0, 1, 2, 5, 6, 7, and 8), both methods achieve exactly the same performance, showing that TSFT can match the accuracy of traditional within-subject decoding in common cases. Two differences are observed: For subject 3, TSFT performs slightly worse (0.8019 vs. 0.8031). For subject 4, TSFT performs much better (0.9625 vs. 0.8969), indicating that selecting another subject’s model can sometimes outperform using the subject’s own model. On average, TSFT achieves a higher F1 score than the within-subject approach (0.7672 vs. 0.7601). This suggests that the model-selection mechanism in TSFT has the potential to improve decoding performance by choosing a more suitable model based on data similarity. Although the improvement mainly comes from one subject, this result demonstrates that TSFT can provide performance gains in certain cases while maintaining overall reliability.

**TABLE V:** Comparison of F1 Scores of Two Different Decoding Paradigms

SubjectsParadigms	Within-subject decoding paradigm	TSFT decoding paradigm
0	0.6506	0.6506
1	0.7064	0.7064
2	0.8375	0.8375
3	0.8031	0.8019
4	0.8969	0.9625
5	0.7747	0.7747
6	0.8406	0.8406
7	0.7844	0.7844
8	0.5465	0.5465
Average	0.7601	0.7672

### B. ERN Dataset

Table VI reports the distribution distances between test data and training data for 16 subjects in the ERN dataset. Each row corresponds to a test subject, and each column corresponds to a training subject. The smaller the number, the more similar the data distributions, and the more suitable that subject’s model is for decoding. Unlike the previous dataset, this table shows that most subjects are not best matched with their own training data. Only subjects 9 and 14 select their own models as the closest match. For the majority of subjects, the training data of other subjects has a smaller distance than their own training data, indicating much stronger cross-subject similarity and variability. This makes the ERN dataset a more challenging and realistic test case for TSFT, because model selection is no longer trivial. The subject authentication module must work harder to identify a good match among many candidates rather than defaulting to each subject’s own model. Overall, this table demonstrates that subject variability in this dataset is much stronger and more irregular, making it an important complement to the earlier dataset and a more meaningful test of TSFT’s robustness.

**TABLE VI:** Distribution Distances Between Test Data and Train Data and the Matching Results

Test dataTrain data	0	1	2	3	4	5	6	7	8	9	10	11	12	13	14	15
0 $\qquad \rightarrow$ 4	74.85	13.86	37.51	264.7	7.83	222.4	293.33	32.41	230.04	110.28	35.52	174.21	24.52	259.88	115.55	247.27
1 $\qquad \rightarrow$ 0	1.42	20.15	7.02	13.38	50.83	7.91	21.8	90.56	6.0	22.85	290.96	670.11	144.54	11.32	5.71	18.76
2 $\qquad \rightarrow$ 0	2.31	42.59	7.24	71.02	8.66	38.78	70.83	24.67	48.01	9.71	158.98	472.85	51.94	60.99	13.94	45.26
3 $\qquad \rightarrow$ 6	28.3	3.12	30.49	3.11	95.54	5.56	0.31	149.2	1.51	44.7	434.9	912.33	229.23	5.1	4.03	0.82
4 $\qquad \rightarrow$ 2	5.16	27.33	2.52	59.47	8.71	26.44	53.66	29.8	36.93	3.42	184.3	525.4	63.52	47.6	12.7	30.76
5 $\qquad \rightarrow$ 2	3.58	5.75	2.94	27.52	31.43	5.2	18.44	66.11	11.75	8.5	276.4	677.7	118.57	17.84	8.99	7.08
6 $\qquad \rightarrow$ 3	27.77	7.52	33.2	0.41	106.61	6.93	0.49	165.01	1.08	53.32	449.88	923.22	244.56	1.54	3.42	4.23
7 $\quad \, \rightarrow$ 13	15.1	7.38	20.3	2.92	86.29	2.81	2.47	140.46	0.39	40.66	398.78	841.55	210.24	0.35	0.88	6.64
8 $\qquad \rightarrow$ 0	1.94	19.09	7.4	11.24	54.26	7.11	19.46	95.86	4.55	25.5	301.25	684.95	150.18	9.55	6.74	17.51
9 $\qquad \rightarrow$ 9	83.94	92.14	69.6	190.75	30.25	75.45	160.81	19.22	126.13	5.23	146.72	508.63	47.93	172.85	118.96	110.16
10 $\qquad \rightarrow$ 9	8.7	31.4	8.1	72.16	5.49	33.74	62.66	23.21	46.42	2.35	171.34	515.42	58.11	57.6	18.18	38.22
11 $\quad \, \rightarrow$ 10	184.92	50.37	132.44	463.24	66.13	391.19	484.33	49.73	411.23	129.19	8.56	66.9	19.82	450.04	255.39	412.85
12 $\qquad \rightarrow$ 1	187.53	26.36	103.18	450.35	59.52	398.18	491.04	77.28	407.14	185.82	30.66	70.81	46.04	446.86	247.26	430.51
13 $\qquad \rightarrow$ 3	26.39	10.8	33.72	0.28	110.15	7.83	1.92	170.29	0.95	56.18	450.17	916.29	248.6	0.54	3.25	7.4
14 $\quad \, \rightarrow$ 14	11.86	2.21	13.88	6.9	66.76	0.56	2.99	114.62	2.24	27.12	364.9	805.23	182.12	3.51	0.36	2.73
15 $\qquad \rightarrow$ 6	20.46	3.41	22.65	2.97	87.0	3.07	0.82	139.12	1.55	39.43	411.13	869.07	213.09	2.76	2.56	1.99

Table VII shows the time required for the subject authentication module to compute distribution distances and select the appropriate model for each subject in the ERN dataset. Each entry represents one matching case (e.g., “0 $\rightarrow$ 4” means subject 0 is matched to model 4), and the value indicates how long the selection process takes in microseconds. The time ranges from 116.7 $\mathrm{\mu }$s to 214.0 $\mathrm{\mu }$s, with an average of 130.2 $\mathrm{\mu }$s. These results show that the model selection step is extremely fast and introduces negligible delay, making it suitable for real-time EEG decoding scenarios.

**TABLE VII:** Time Spent for Calculating Distribution Distances and Finding Matches (Unit: Microseconds)

0 $\rightarrow$ 4	1 $\rightarrow$ 0	2 $\rightarrow$ 0	3 $\rightarrow$ 6	4 $\rightarrow$ 2	5 $\rightarrow$ 2	6 $\rightarrow$ 3	7 $\rightarrow$ 13	8 $\rightarrow$ 0	9 $\rightarrow$ 9	10 $\rightarrow$ 9	11 $\rightarrow$ 10	12 $\rightarrow$ 1	13 $\rightarrow$ 3	14 $\rightarrow$ 14	15 $\rightarrow$ 6	Average
214.0	134.5	125.6	122.1	121.2	119.9	120.3	118.0	116.7	119.7	118.2	117.6	175.4	125.3	117.4	117.3	130.2

Table VIII compares the decoding performance (F1 score) between the within-subject decoding paradigm and the TSFT decoding paradigm on the ERN dataset. For subjects 9, 12, and 14, both approaches achieve exactly the same results, indicating that TSFT can reproduce the performance of traditional within-subject decoding. For the remaining subjects, the F1 scores differ, and no single method consistently dominates across all subjects. TSFT performs better for some subjects (e.g., subjects 0, 3, 4, 5, and 11), while within-subject decoding performs better in others (e.g., subjects 1, 2, 6, 8, 13, and 15). Overall, the average F1 scores are nearly identical: 0.7572 for within-subject decoding and 0.7568 for TSFT, resulting in only a negligible difference of 0.0004. This close result shows that TSFT maintains the accuracy of within-subject decoding while enabling model reuse across users. These results demonstrate that TSFT can achieve comparable performance without requiring subject-specific training, confirming the effectiveness of the subject authentication module and the reliability of the distribution-based model selection strategy.

**TABLE VIII:** Comparison of F1 Scores of Two Different Decoding Paradigms

SubjectsParadigms	Within-subject decoding paradigm	TSFT decoding paradigm
0	0.7179	0.7424
1	0.9418	0.9290
2	0.9451	0.9405
3	0.7338	0.7483
4	0.5691	0.6074
5	0.5952	0.6512
6	0.6528	0.6099
7	0.6849	0.6846
8	0.7600	0.7417
9	0.8439	0.8439
10	0.7805	0.7805
11	0.9000	0.9189
12	0.9529	0.9529
13	0.7500	0.7123
14	0.6757	0.6757
15	0.6116	0.5693
Average	0.7572	0.7568

### C. P300-LINI Dataset

We selected the P300-LINI dataset because, unlike the previous two datasets with predefined training and test splits, it allows flexible shuffling to create diverse splits. This enables multiple independent cases for comparing TSFT and within-subject decoding, providing a more comprehensive analysis. We used $RepeatedStratifiedKFold$ to generate these splits. The experimental results also differ from the earlier datasets. First, distance results are not shown, as they are three-dimensional and too extensive to present effectively. Second, performance results for within-subject and TSFT decoding are presented separately, as each is two-dimensional and cannot be combined into a single table.

Table IX reports the F1 scores of the within-subject decoding paradigm evaluated on a dataset consisting of 22 subjects. Each row corresponds to one subject (Subjects 0–21), and each column from 0 to 14 represents results from one experimental round generated by the $RepeatedStratifiedKFold$ procedure with 5 folds and 3 repetitions (15 rounds in total). Each entry is the F1 score obtained when a model trained on one subject is evaluated using that same subject’s test data. The bottom row (“Average”) shows the average F1 score across all subjects for each round. The most important figure is the “Total Average” shown on the far right: 0.8888, which represents the mean performance across all subjects and all trials. This result closely matches the previously reported F1 score of about 0.89 for within-subject P300 decoding in the literature [59], serving as a successful replication and validation of the implementation. Overall, the table demonstrates that within-subject decoding achieves high and consistent performance across both subjects and experimental runs, establishing a reliable baseline for comparison with TSFT.

**TABLE IX:** F1 Scores of Within-Subject Decoding Paradigm

SubjectsSplits Repeats	0	1	2	3	4	5	6	7	8	9	10	11	12	13	14	Total Average
0	0.9099	0.9417	0.9107	0.9124	0.9096	0.9161	0.9008	0.9293	0.9061	0.8890	0.9269	0.9102	0.9379	0.9098	0.9105	0.8888
1	0.9519	0.9069	0.9242	0.9461	0.9173	0.9228	0.9314	0.9330	0.9432	0.9055	0.9202	0.9394	0.9099	0.9267	0.9321	
2	0.8999	0.9080	0.8979	0.9184	0.8874	0.8993	0.9291	0.9024	0.9143	0.8967	0.9152	0.8955	0.9141	0.8955	0.9144	
3	0.6421	0.7090	0.7417	0.7924	0.7426	0.7501	0.7333	0.7235	0.7469	0.7423	0.7222	0.7282	0.7779	0.7415	0.7056	
4	0.8298	0.8577	0.8667	0.8490	0.8782	0.8834	0.8770	0.7746	0.8669	0.8358	0.8381	0.8692	0.8382	0.8644	0.8341	
5	0.9450	0.9413	0.9529	0.9433	0.9562	0.9543	0.9274	0.9505	0.9525	0.9374	0.9536	0.9388	0.9562	0.9427	0.9365	
6	0.8119	0.7984	0.8308	0.8446	0.8234	0.7953	0.8455	0.8424	0.8170	0.8240	0.8360	0.8063	0.8051	0.8605	0.8422	
7	0.8657	0.8275	0.8561	0.8623	0.8541	0.8495	0.8661	0.8250	0.8567	0.8675	0.8971	0.8265	0.8501	0.8558	0.8413	
8	0.9074	0.9264	0.9153	0.8983	0.8961	0.9112	0.9096	0.8759	0.9129	0.9048	0.8980	0.9061	0.9073	0.9214	0.8970	
9	0.9118	0.9047	0.9069	0.9020	0.8892	0.8914	0.9019	0.9050	0.9107	0.9320	0.9218	0.8780	0.9202	0.9043	0.8919	
10	0.8718	0.8664	0.8924	0.8995	0.8788	0.8255	0.9029	0.8771	0.8532	0.8747	0.8714	0.8707	0.8747	0.8901	0.8567	
11	0.8765	0.8804	0.8906	0.8882	0.9084	0.8904	0.8789	0.9004	0.8676	0.8805	0.8968	0.8518	0.8949	0.8874	0.9024	
12	0.8761	0.8594	0.8815	0.8658	0.8471	0.8791	0.8727	0.8694	0.8456	0.8492	0.8841	0.8571	0.8593	0.8704	0.8880	
13	0.9235	0.8645	0.8923	0.8994	0.9081	0.9208	0.8703	0.9133	0.8566	0.9240	0.9130	0.9017	0.8712	0.8959	0.9044	
14	0.8775	0.8682	0.8469	0.8549	0.8616	0.8609	0.8385	0.8736	0.8179	0.8593	0.8812	0.8292	0.8692	0.8308	0.9146	
15	0.9378	0.9189	0.9157	0.9363	0.9194	0.9326	0.9194	0.9051	0.9501	0.8778	0.9124	0.9661	0.9171	0.9158	0.9117	
16	0.8235	0.8071	0.8500	0.8556	0.8128	0.8141	0.8415	0.8571	0.8475	0.8082	0.8529	0.8411	0.8404	0.8138	0.8192	
17	0.9700	0.9845	0.9675	0.9703	0.9638	0.9751	0.9676	0.9568	0.9709	0.9715	0.9745	0.9563	0.9695	0.9733	0.9627	
18	0.9445	0.9358	0.9395	0.9347	0.9506	0.9636	0.9246	0.9330	0.9252	0.9522	0.9393	0.9287	0.9445	0.9428	0.9522	
19	0.9113	0.9483	0.9617	0.9392	0.9346	0.9098	0.9362	0.9379	0.9602	0.9385	0.9162	0.9306	0.9553	0.9426	0.9571	
20	0.9276	0.9467	0.9455	0.9239	0.9196	0.9503	0.9650	0.9309	0.9144	0.9305	0.9268	0.9350	0.9388	0.9163	0.9470	
21	0.8623	0.8925	0.8823	0.8475	0.8226	0.8454	0.8516	0.8789	0.8637	0.8649	0.8568	0.8949	0.8537	0.8658	0.8815	
Average	0.8854	0.8861	0.8940	0.8947	0.8855	0.8882	0.8905	0.8861	0.8864	0.8848	0.8934	0.8846	0.8912	0.8894	0.8910	

Table X presents the F1 scores of the TSFT decoding paradigm on the same dataset. Similar to the previous Table IX, each row corresponds to one subject (Subjects 0–21), and the columns from 0 to 14 represent the results from 15 evaluation rounds generated using $RepeatedStratifiedKFold$ with 5 folds and 3 repetitions. Each entry reports the F1 score achieved when the test data are decoded by the model selected from the pool based on the subject authentication mechanism. The bottom row (“Average”) shows the average F1 score across all subjects for each round. The most important value is the “Total Average”, shown on the right: 0.8804. This result is only slightly lower than the within-subject decoding average of 0.8888, with a difference of less than 0.01. This indicates that despite not using subject-specific calibration, TSFT achieves performance comparable to traditional within-subject decoding. These results demonstrate that TSFT can maintain strong decoding accuracy while eliminating the need for retraining on each new subject. The small performance gap highlights a favorable trade-off between accuracy and usability, confirming TSFT as a practical and scalable alternative to calibration-based approaches.

**TABLE X:** F1 Scores of TSFT Decoding Paradigm

SubjectsSplits Repeats	0	1	2	3	4	5	6	7	8	9	10	11	12	13	14	Total Average
0	0.9099	0.9417	0.9107	0.9124	0.9096	0.9161	0.9008	0.9293	0.9061	0.8890	0.9269	0.9102	0.9379	0.9098	0.9105	0.8804
1	0.9519	0.9069	0.9242	0.9461	0.9173	0.9228	0.9314	0.9330	0.9432	0.9055	0.8331	0.9394	0.9099	0.9267	0.9321	
2	0.8999	0.9080	0.8979	0.9184	0.8874	0.8993	0.9291	0.9024	0.8430	0.8967	0.9152	0.8955	0.9141	0.8955	0.9144	
3	0.6421	0.7090	0.7417	0.7924	0.7426	0.7501	0.7333	0.7235	0.7469	0.7423	0.7222	0.7282	0.7779	0.7415	0.7056	
4	0.8298	0.8577	0.8667	0.8490	0.8782	0.8834	0.8770	0.7746	0.8669	0.8358	0.8381	0.8692	0.8382	0.8644	0.8341	
5	0.9450	0.9413	0.9529	0.9433	0.9562	0.9543	0.9274	0.9505	0.9525	0.9374	0.9536	0.9388	0.9562	0.9427	0.9365	
6	0.8119	0.7984	0.8308	0.8446	0.8234	0.7953	0.8455	0.7568	0.8170	0.8240	0.8360	0.8063	0.8051	0.8605	0.8422	
7	0.8657	0.8275	0.8561	0.8623	0.8541	0.8495	0.8661	0.8250	0.8567	0.8675	0.8971	0.8265	0.8501	0.8558	0.8413	
8	0.9074	0.9264	0.9153	0.8983	0.8961	0.9112	0.9096	0.8759	0.9129	0.9048	0.8980	0.9061	0.9073	0.9214	0.8970	
9	0.9118	0.9047	0.9069	0.9020	0.8892	0.8914	0.9019	0.9050	0.9107	0.9320	0.8848	0.8780	0.9202	0.9043	0.8919	
10	0.8718	0.8664	0.8924	0.7848	0.8788	0.8255	0.9029	0.8771	0.8532	0.7795	0.8714	0.8707	0.7721	0.8024	0.6991	
11	0.8765	0.8804	0.8906	0.8882	0.9084	0.8904	0.8789	0.9004	0.8676	0.8805	0.8968	0.8518	0.8949	0.8874	0.9024	
12	0.6660	0.7518	0.8815	0.8658	0.8471	0.7090	0.8727	0.8694	0.7167	0.8492	0.8841	0.8571	0.7135	0.8704	0.8880	
13	0.9235	0.8645	0.8923	0.8994	0.9081	0.9208	0.8703	0.9133	0.8566	0.9240	0.9130	0.9017	0.8712	0.8959	0.9044	
14	0.8775	0.7991	0.8469	0.6476	0.8616	0.7497	0.7976	0.8736	0.8072	0.8593	0.6114	0.8292	0.8692	0.7523	0.6979	
15	0.9378	0.9189	0.9157	0.9363	0.9194	0.9326	0.9194	0.9051	0.9501	0.8778	0.9124	0.9661	0.9171	0.9158	0.9117	
16	0.8235	0.8071	0.8500	0.8556	0.8128	0.8141	0.8415	0.8571	0.8475	0.8082	0.8529	0.8411	0.8404	0.8138	0.8192	
17	0.9700	0.9845	0.9675	0.9703	0.9638	0.9751	0.9676	0.9568	0.9709	0.9715	0.9745	0.9563	0.9695	0.9733	0.9627	
18	0.9445	0.9358	0.9395	0.9347	0.9506	0.9636	0.9246	0.9330	0.9252	0.9522	0.9393	0.9287	0.9445	0.9428	0.9522	
19	0.9113	0.9483	0.9617	0.9392	0.9346	0.9098	0.9362	0.9379	0.9602	0.9385	0.9162	0.9306	0.9553	0.9426	0.9571	
20	0.9276	0.9467	0.9455	0.9239	0.9196	0.9503	0.9650	0.9309	0.9144	0.8562	0.9268	0.9350	0.9388	0.9163	0.9470	
21	0.8623	0.8925	0.8823	0.8475	0.8226	0.8454	0.8516	0.8789	0.8637	0.8649	0.8568	0.8949	0.8537	0.8031	0.8815	
Average	0.8758	0.8781	0.8940	0.8801	0.8855	0.8754	0.8887	0.8822	0.8768	0.8771	0.8755	0.8846	0.8799	0.8790	0.8740	

Table XI reports the time required by the subject authentication module to compute distribution distances and select the most appropriate model for each subject in the TSFT framework. The table includes 22 subjects (rows) and 15 experimental rounds (columns), generated using RepeatedStratifiedKFold with 5 splits and 3 repetitions. Each cell records the model-selection time in microseconds for one subject in one evaluation round. The bottom row (“Average”) shows the mean time across all subjects for each round. Overall, a total of 330 selection measurements are recorded. The most important result is the “Total Average” shown in the bottom-right corner:90.0 $\mathrm{\mu }$s. This extremely small latency demonstrates that the TSFT selection mechanism introduces negligible overhead, making it fully suitable for real-time operation. Combined with the inference time 0.03 s of the P300MCNN, as shown in Table II), these results show that TSFT is computationally efficient and practical for real-world EEG systems.

**TABLE XI:** Time Spent for Calculating Distribution Distances and Finding Matches for Each Split and Repeat Round (Unit: Microseconds)

SubjectsSplits Repeats	0	1	2	3	4	5	6	7	8	9	10	11	12	13	14	Total Average
0	137.6	121.8	121.5	120.9	119.3	122.2	116.8	121.0	117.4	119.4	120.0	116.0	128.6	118.9	115.7	90.0
1	92.9	89.4	89.5	91.2	88.9	87.9	87.8	89.3	89.0	88.2	89.8	87.3	99.8	88.1	85.6	
2	86.8	85.9	84.9	84.3	87.3	84.1	98.1	97.1	83.8	85.3	107.8	83.3	97.9	84.3	81.6	
3	85.6	84.7	83.2	94.5	83.1	82.4	105.8	82.0	107.9	83.0	89.3	101.2	97.0	83.3	79.9	
4	84.8	83.7	82.0	80.4	82.0	81.0	172.6	85.5	84.7	85.3	84.2	97.2	96.8	81.7	78.7	
5	86.2	83.3	82.2	80.4	82.1	82.6	113.7	81.1	82.9	96.4	96.6	97.2	98.5	83.6	80.5	
6	84.9	83.9	82.5	79.6	82.5	82.6	117.7	82.8	82.0	80.7	81.7	97.2	97.3	82.7	99.0	
7	84.4	82.7	81.3	80.3	81.5	81.7	79.9	106.3	81.4	80.3	80.2	96.1	96.5	81.4	79.1	
8	83.9	82.6	81.9	80.1	82.5	81.6	79.5	85.6	82.4	120.5	80.8	96.5	96.2	82.1	79.1	
9	84.6	101.8	83.2	79.6	82.2	82.2	80.3	84.0	82.3	81.3	81.6	97.0	95.8	82.1	78.9	
10	83.0	81.6	82.7	80.9	82.9	84.4	79.9	82.5	80.9	83.0	101.0	97.0	97.8	87.1	80.6	
11	83.0	80.8	84.3	104.7	81.7	82.3	80.8	82.1	80.1	103.8	85.9	96.3	119.2	151.5	106.0	
12	87.8	82.7	82.2	84.1	81.8	83.5	81.2	93.5	81.7	88.3	81.9	101.0	99.5	85.5	87.1	
13	109.8	106.0	82.0	81.7	118.1	124.4	80.2	82.6	102.4	83.1	81.7	96.4	104.0	82.8	81.6	
14	89.0	87.8	93.8	84.3	85.8	86.8	81.5	82.4	86.0	81.5	82.8	96.5	86.7	84.5	82.8	
15	86.7	100.3	80.2	99.0	82.6	100.8	153.5	81.8	104.4	81.2	100.6	96.2	83.7	106.9	100.4	
16	85.7	83.4	79.6	83.6	94.7	86.9	85.7	81.2	84.1	81.0	84.6	96.7	83.3	87.2	82.9	
17	99.3	81.1	80.1	83.5	80.7	82.4	82.4	81.3	82.1	81.6	81.9	96.7	82.4	89.3	81.2	
18	84.9	81.3	79.7	81.5	80.2	81.0	81.2	81.8	81.9	81.7	82.0	96.6	81.1	89.1	79.8	
19	82.7	80.3	119.8	80.3	79.7	80.5	80.3	81.4	80.3	80.7	81.0	95.4	91.4	92.6	80.2	
20	82.6	79.8	79.9	79.5	79.9	80.4	80.5	80.4	80.3	82.3	83.3	96.3	96.4	89.9	79.7	
21	82.3	80.3	176.2	79.8	80.0	80.1	80.2	81.2	80.8	104.4	80.7	101.3	96.0	97.7	180.3	
Average	89.5	87.5	90.6	86.1	86.3	87.4	95.4	86.7	87.2	88.8	88.2	97.1	96.6	91.5	90.0	

## V. Conclusion

This study presents TSFT, a calibration-free framework for EEG BCIs that addresses one of the field’s most persistent challenges: repeated subject-specific calibration. By combining compact deep learning models with a data-driven subject authentication module, TSFT enables real-time selection of the most suitable decoding model based solely on the characteristics of incoming EEG signals. This dynamic model-selection mechanism replaces time-consuming calibration and retraining with an efficient, plug-and-play alternative that naturally adapts to subject variability.

Comprehensive evaluations on three public datasets (MI, ERN, and P300) demonstrate the effectiveness of TSFT. Specifically, TSFT outperforms the within-subject baseline on the MI dataset (F1 score: 0.7672 vs. 0.7601), achieves nearly identical performance on the ERN dataset (0.7568 vs. 0.7572), and shows only a marginal decrease on the P300 dataset (0.8804 vs. 0.8888), with both values significantly exceeding OMFA’s performance. These results confirm that TSFT can achieve accuracy comparable to within-subject decoding while completely eliminating the need for calibration. Additionally, the success of signature-based model selection in identifying optimal decoding models across subjects highlights the robustness and adaptability of the proposed framework.

In summary, TSFT provides a practical and scalable solution for calibration-free EEG decoding. By replacing the traditional OMFA paradigm and costly retraining procedures with fast, data-driven model selection, it significantly improves usability and deployment feasibility. This work represents a step toward truly user-friendly EEG-based BCIs and opens the door to more adaptable and personalized neurotechnology systems in real-world environments.

## VI. Future Work

There are mainly two aspects for future work. First is the signature design of each model. The current implementation uses a simple channel-wise standard deviation signature to demonstrate feasibility. Since TSFT’s performance depends on signature quality, future work will explore more advanced designs. Signatures should capture both model and data characteristics, using richer features such as spectral information and EEG-based identity recognition. They should also require minimal input data, ideally one or a few epochs, to improve response speed and practical applicability. The second aspect is to extend TSFT to other neuroimaging modalities beyond EEG, such as fMRI and fNIRS.
